# Disentangling function from topology to infer the network properties of disease genes

**DOI:** 10.1186/1752-0509-7-5

**Published:** 2013-01-16

**Authors:** Dario Ghersi, Mona Singh

**Affiliations:** 1 Lewis-Sigler Institute for Integrative Genomics, Princeton University, Princeton, NJ 08540, USA; 2 Department of Computer Science, Princeton University, Princeton, NJ 08540, USA

**Keywords:** Disease genes, Networks, Functional bias, Gene ontology

## Abstract

**Background:**

The topological features of disease genes within interaction networks are the subject of intense study, as they shed light on common mechanisms of pathology and are useful for uncovering additional disease genes. Computational analyses typically try to uncover whether disease genes exhibit distinct network features, as compared to all genes.

**Results:**

We demonstrate that the functional composition of disease gene sets is an important confounding factor in these types of analyses. We consider five disease sets and show that while they indeed have distinct topological features, they are also enriched in functions that *a priori* exhibit distinct network properties. To address this, we develop a computational framework to assess the network properties of disease genes based on a sampling algorithm that generates control gene sets that are functionally similar to the disease set. Using our function-constrained sampling approach, we demonstrate that for most of the topological properties studied, disease genes are more similar to sets of genes with similar functional make-up than they are to randomly selected genes; this suggests that these observed differences in topological properties reflect not only the distinguishing network features of disease genes but also their functional composition. Nevertheless, we also highlight many cases where disease genes have distinct topological properties even when accounting for function.

**Conclusions:**

Our approach is an important first step in extracting the residual topological differences in disease genes when accounting for function, and leads to new insights into the network properties of disease genes.

## Background

Network biology provides a holistic framework for understanding pathological processes at a system-wide level. By enabling human disorders to be cast within the context of large-scale interactomes, network-based approaches shed light on the roles of genes both within the context of specific diseases as well as across multiple disorders [1]. Further, the network properties of disease genes hold promise for improving candidate gene prioritization in a time and cost effective way, and for helping to identify novel candidates for target-based therapies (for a review, see [2]). The rapid growth of data associating genes to diseases (e.g., mutations catalogued from the Cancer Genome Project [3] or from exome studies of individuals afflicted with a disease of interest), along with the availability of large-scale human interaction data [4-6], provides further impetus for developing and applying network-based approaches for analyzing disease genes.

Computational analysis of genes, both disease and non-disease, within interactomes has demonstrated that the topological network features of their corresponding proteins reveal important aspects of their underlying functioning (reviews, [7,8]). Groups of interacting proteins work together in modules to achieve specific biological functions [9-14]. Simple topological measures can reflect important cellular properties; for example, proteins with high degree centrality, or a large number of interactions, are more likely to be evolutionarily conserved and essential to the survival of the cell than other proteins [15-17]. In the context of human diseases, genes associated with specific disorders tend to cluster in physical interaction networks [1,18], suggesting that these diseases result from the malfunctioning of specific functional modules. Cancer mutated genes have been found to be enriched in their number of physical interactions [19,20], whereas genes with mutations responsible for inherited disorders have a less clearly defined position in the network. More specifically, an early study showed that inherited disease genes are characterized by larger than expected degree [21], but it has been since argued that this observation is due to the correlation between essentiality and degree, and that once the small fraction of disease genes that are also essential are removed from consideration, inherited disease genes do not tend to have a higher number of interactions [1]. In a more recent study, genes involved in inherited and complex diseases have been found to have higher global centrality, as measured by betweenness centrality, and to occur in less dense portions of the network, as measured by clustering coefficient, than non-disease genes; it has been proposed that these disease genes play an important role in bridging otherwise disconnected parts of the interactome [22].

While these numerous studies have found that various disease gene sets have distinct topological network features, and have argued that these features reflect important aspects of pathology, here we aim to uncover whether network features reflect functional composition, and if so, to develop and apply a computational approach that controls for this. As a first step, we perform a comprehensive analysis of the topological network properties of five different disease sets, including genes implicated in monogenic or polygenic Mendelian disorders, or mutated, over-expressed or under-expressed in cancer. We begin with the observation that these five disease sets are enriched in specific functional categories. Further, we demonstrate that genes associated with specific functional categories themselves tend to have distinct topological properties *a priori*. Together, these two findings raise the possibility that a significant portion of the observed topological differences between disease genes and other genes may in fact be due to differences in their functional compositions. If this is the case, then the topological properties of disease genes may contribute only a limited amount of new information beyond what is already implied by their functional make-up, thereby lessening their impact in furthering our understanding of disease pathology or in uncovering new disease genes.

We propose that in order to assess whether a set of disease genes is topologically distinct from other genes, it is critically important to explicitly consider biological function. Towards this end, we develop a sampling strategy to generate control sets of genes functionally similar to a reference set. We apply our sampling approach to compare each of the disease sets to control sets of genes with similar functional compositions. In contrast to previous studies that consider and compare just the medians or means of topological properties, we compare the distributions of these properties between disease and non-disease gene sets over their interquartile ranges. We find that in most cases considered, the topological properties of disease genes appear less distinct when compared to functionally similar groups of genes than when considering randomly selected genes; that is, at least some of the observed differences in the topological properties of disease genes, as compared to all genes, is in fact explained by their specific functional compositions. For example, inherited disease genes have previously been found to have a significantly lower clustering coefficient than randomly selected genes [22], but we find more typical or even higher values when considering groups of functionally similar genes. Statistically significant differences do emerge, however, in some of the topological properties of disease sets, even when considering function, and different disease sets can exhibit opposing trends. For example, genes over-expressed in cancer tend to have, on average, higher interaction degree, higher clustering coefficient, and higher betweenness centrality than functionally similar sets of genes, but these trends are reversed for cancer under-expressed genes. Overall, using our sampling approach, we show that the functional composition of a disease set is an important factor in its observed topological properties, but that the broad disease group (e.g., Mendelian disorders vs. cancer) and the type of involvement (e.g., mutation vs. altered expression in cancer) play significant roles as well.

## Results

### Preliminaries

We analyze the functional enrichment and network features of five sets of disease genes: (1) genes implicated in Mendelian disorders, as reported in the OMIM catalogue [23] (full OMIM); (2) the subset of OMIM genes involved in monogenic disorders, derived from Cai et al. [22] (monogenic); (3) genes with mutations reported in more than one cancer type, derived from the IntOGen database [24] (cancerMut); (4) genes significantly over-expressed in cancers (from IntOGen) (cancerOverExpr) and (5) genes significantly under-expressed in cancers (from IntOGen) (cancerUnderExpr).

In the main body of the paper, we focus on a single network and set of functional terms, but also consider other networks and functional terms in the Supplement. Specifically, our primary analysis uses a subset of Gene Ontology (GO) biological process terms that correspond to broad functional classes (“Informative Terms” [25], see Materials and Methods for more details). Further, we perform our primary network analysis on a comprehensive human protein-protein interaction network [26], and limit our analysis to proteins annotated by at least one informative term. We consider three widely studied network properties: degree centrality, betweenness centrality and clustering coefficient. A protein’s degree centrality is the number of binding partners it has, whereas its betweenness centrality is defined as the sum over each pair of proteins of the fraction of the shortest paths between them that pass through it [27]; these are, respectively, local and global measures of the topological prominence of a protein. The clustering coefficient of a protein measures the tendency of its binding partners to also bind to each other [27]; it is a local measure of the network density of a protein’s neighborhood.

### Disease gene sets are topologically distinct from each other and from the set of all genes

**Figure 1:**
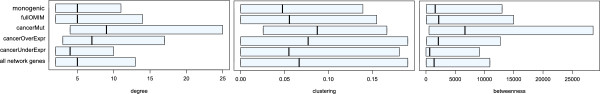
**Disease sets are topologically distinct from each other.** The 25th-75th percentile of degree (left), clustering coefficient (middle) and betweenness centrality (right) values for each of the five disease sets (see text) and the set of all annotated genes. Medians are indicated by the vertical bar, with the 2nd and 3rd interquartile ranges shown via the boxes to the left and the right of the median.

We begin by observing that the five different disease sets exhibit starkly different topological properties (Figure 1). Their median degrees range from 4 for cancer under-expressed genes to 9 for cancer mutated genes. Cancer mutated genes have a median degree 1.8 times larger than that of OMIM genes and the monogenic subset (*p*<2*.*2*e*−16, Wilcoxon’s rank sum test), and more than twice that of the cancer under-expressed genes (*p*<2*.*2*e*−16). The median clustering coefficient ranges from 0.048 for genes involved in monogenic disorders to 0.077 for cancer over-expressed genes and 0.087 for cancer mutated genes. Both cancer over-expressed and cancer mutated genes are significantly more clustered than the genes in all the other disease sets (all *p*-values <0*.*005). Cancer mutated genes have a median betweenness centrality value that is over three times the medians of all the other disease sets (all *p*-values <1*e*−9), and is ten times that of cancer under-expressed genes. Overall, the observed variation in topological properties between various disease sets is consistent with the apparent lack of agreement in previous studies that each focused only on specific groups of disorders [1,18-20,22].

### Disease gene sets show characteristic functional compositions

**Figure 2:**
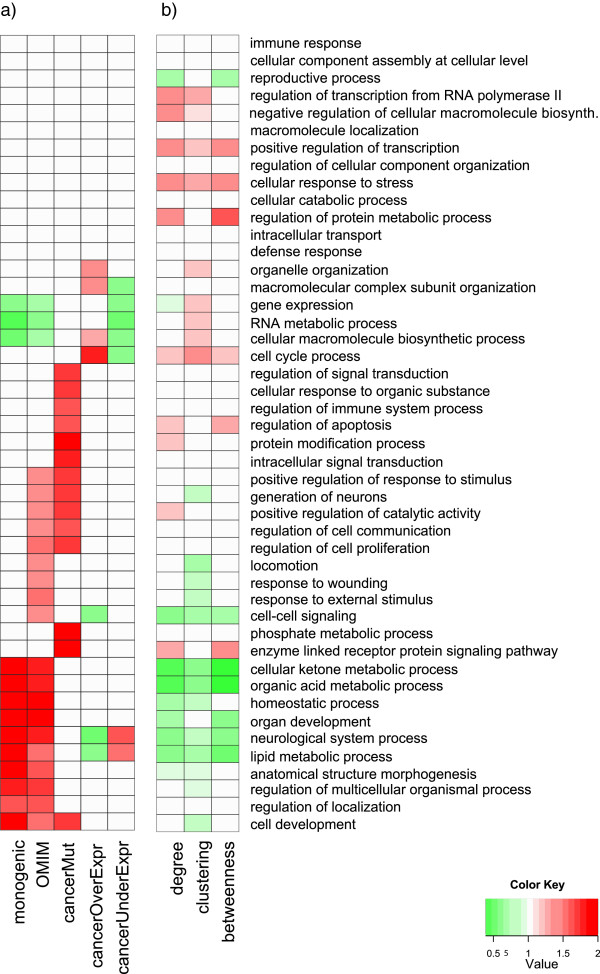
**Function, network topology and disease are interrelated. (a)** In each disease set, significantly enriched and under-enriched functions are shown in red and green, respectively (*p*<0*.*05, Bonferroni-corrected hypergeometric test). The fold enrichment or depletion for each disease set and term is plotted using a red-green gradient and calculated as the ratio of the fraction of genes in the disease set that are annotated by that term to the fraction of all annotated genes that include that term. **(b)** For each of the three topological properties and each term, we compared the distributions of the topological property restricted to genes annotated with that term versus all annotated genes. Red (green) depicts a significant difference as determined via Wilcoxon’s rank sum test (Bonferroni-corrected *p*-value <0*.*05); that is, genes having this functional term differ in this topological property from the set of all annotated genes. We use a red-green gradient to plot the ratio of the average of the property in the genes annotated with the term to the average of the property in all annotated genes; however, in order not to overweigh the contribution of genes with multiple annotations, when computing the average of the property across genes annotated with a particular term, we weigh the contribution of each gene inversely to the number of terms with which it is annotated.

Having highlighted the significant topological differences in the five disease sets, we next investigate their functional compositions. For each of the disease sets, we calculate the GO functional enrichment as compared to the full human interactome, using the hypergeometric test (Figure 2). As expected, all disease sets contain significantly over- or under-enriched functional terms (Bonferroni corrected *p*-values <0*.*05), and therefore none of them represents a functionally unbiased sample of the human interactome. This simple GO enrichment analysis broadly recapitulates crucial aspects of the various types of diseases. For example, Mendelian disorder genes (both monogenic and full OMIM sets) are found to be enriched in developmental and metabolic terms (e.g., organ development, anatomical structure morphogenesis, organic acid metabolic process, lipid metabolic process), as one would expect for diseases that are largely congenital or early-onset. In contrast, genes involved in cancer show different patterns of enrichment, depending upon whether they are somatically mutated, over-expressed or under-expressed: cancer mutated genes show a significant involvement in signaling and apoptosis (e.g., intracellular signal transduction and regulation of apoptosis), while genes significantly over-expressed in cancers are enriched in cell cycle terms. Finally, the set of genes significantly under-expressed in cancer are depleted of the biological processes that are most critical to rapidly dividing cells (e.g. RNA metabolic process, cell cycle). A comprehensive enrichment analysis subdivided by anatomical region for cancer over-expressed and under-expressed genes is provided in Additional file 1 Figures S7 and S8, and shows that distinct functional enrichments are also apparent in more fine-grained definitions of disease sets.

### The functions enriched in the disease sets are associated with distinct topological properties *a priori*

Given the characteristic functional compositions exhibited by the disease sets, we next address the following question: are genes annotated with these functions topologically distinct from the rest of the interactome *a priori*? To address this, for each informative term we calculate the average degree, clustering coefficient and betweenness centrality of the genes annotated with the term over the average of all annotated genes (Figure 2b). Several terms annotate genes whose topological properties deviate significantly from the background (Bonferroni corrected *p*<0*.*05, Wilcoxon’s rank sum test). A subset of these terms are also significantly enriched (or under-enriched) in disease sets (Figure 2a).

### A novel sampling approach allows a functionally constrained comparison of disease and control sets

The results presented above show that disease genes tend to be enriched in or depleted of specific functions, in a disease set dependent manner, and that many of these functions are also associated with topological properties that deviate significantly from their values in the full interactome. This suggests that the functional make-up of disease genes represents a confounding factor when studying their topological properties. Do the previously observed topological differences in disease genes simply reflect their functional composition? If this is the case, then disease genes are topologically indistinguishable from the rest of the genes in their functional modules.

In order to address this question, we develop a procedure to minimize the influence of functional composition in gene sets. The idea behind the approach (described in more details in Materials and Methods) is to randomly sample a set of “control” genes from a background set of genes lacking the property that defines “disease genes,” such as mutation or altered expression in cancer. The set of sampled genes has the same size as the disease set and our goal is that it should also have a similar functional composition. We note that this is a challenging computational task because genes can have multiple annotations. We call the samples generated by our procedure “function-constrained” and we refer to the samples simply drawn from the control pool without regard for their functional composition as “unconstrained” samples.

**Figure 3:**
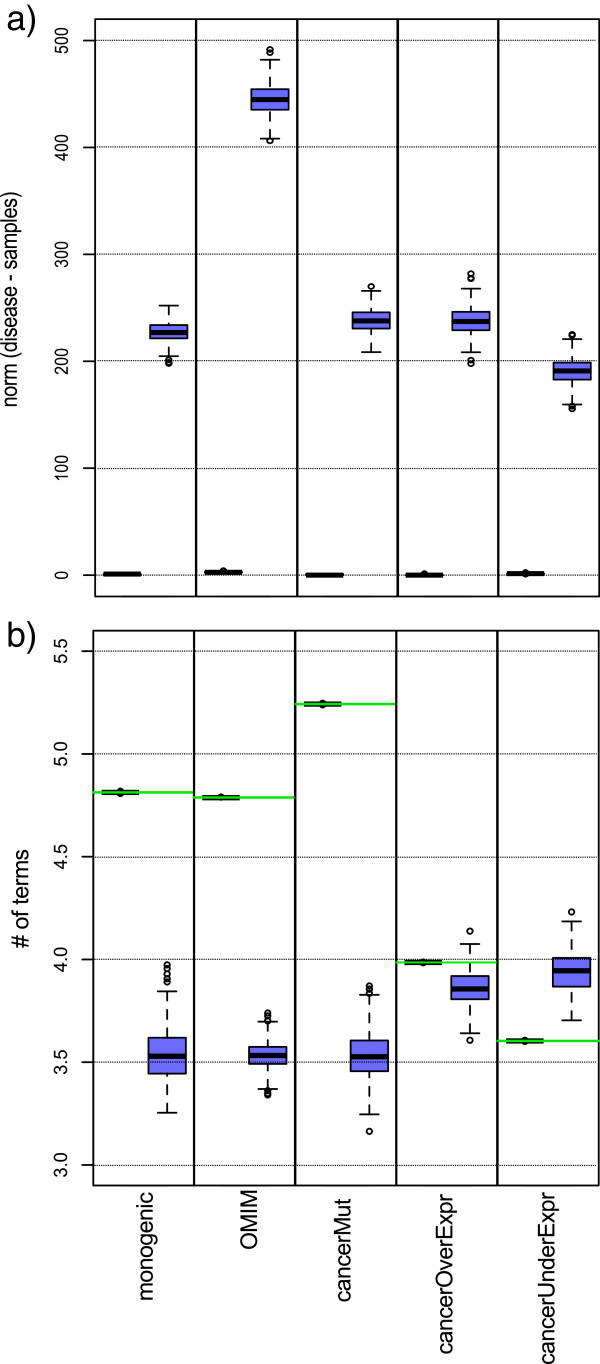
**Function-constrained samples closely match the functional composition and number of terms per protein of disease sets. (a)** Boxplots show the Euclidean norm between the distribution of informative term functional annotations in the disease set versus those found in the samples; this corresponds to the square root of the functional distance defined in the Methods. Function-constrained samples are shown in the left columns and the unconstrained samples are shown in the right columns. The functional distribution of the function-constrained samples are close to the target distribution in all samples. **(b)** Distribution of the average number of terms per protein in the function-constrained samples (left columns) and unconstrained samples (right columns, blue). The average number of terms in each disease set is shown as a horizontal green line. Function-constrained samples have similar average number of terms per protein as the disease sets, whereas the unconstrained samples differ substantially.

To test our sampling procedure, for each target disease set we generate 1000 function-constrained samples and 1000 unconstrained samples. We compare the distribution of informative terms in the samples against the distribution observed in the disease sets. We find that our sampling procedure effectively reduces the differences between the functional composition of the disease set and that of the function-constrained samples (Figure 3a); in contrast, we observe a substantial deviation of the per-term distribution in the unconstrained samples as compared to the target distribution arising from the disease set.

We also consider the average number of terms per gene in the function-constrained and unconstrained samples, and compare it with the average number of terms per gene in the disease sets. We find that function-constrained samples closely match the average number of terms per gene observed in disease sets (Figure 3b), whereas unconstrained samples show very different values. Comparing the results across the five disease sets, cancer mutated and Mendelian disorders genes have on average a higher number of terms per gene than randomly selected sets of genes, whereas cancer under-expressed genes have on average a lower number of terms. This issue of “multifunctionality” is important because of its direct relationship with degree. In agreement with the observation of Gillis and Pavlidis [28], we find that multiply annotated (“multifunctional”) genes tend to be more central. For example, in our network, the median number of informative terms per gene is 3, and whereas the median degree of genes annotated with more than 3 terms is 9, it is only 4 for genes with less than 3 terms (*p*<2*.*2*e*−16, Wilcoxon’s rank sum test).

To ensure that our sampling approach yields sufficiently variable samples, we also quantify the overlap between samples by computing the fraction of genes shared by an increasing number of samples, using the “monogenic” set as an example. While the unconstrained samples exhibit higher variability than the function-constrained samples (as expected), for both approaches, the fraction of genes shared between all samples approaches 0 as the number of samples is increased (Additional file 1 Figure S1). Thus, our approach successfully reduces the functional biases present in samples as compared to the disease set while at the same time yielding diverse samples.

### The functional composition of disease sets partially accounts for their topological properties

**Figure 4:**
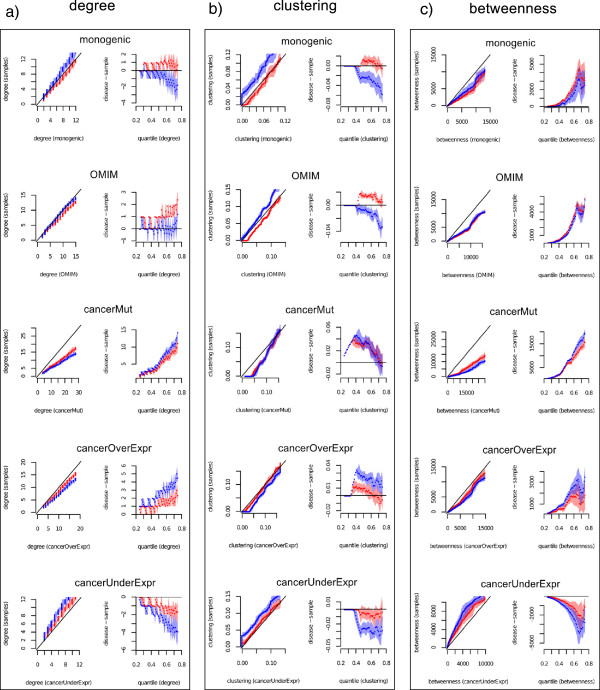
**Disease genes show distinct topological features even when considering function.** Q-Q plots (left-most columns) and difference plots (right-most columns) for degree **(a)**, clustering coefficient **(b)** and betweenness centrality **(c)**. The Q-Q plots are obtained by plotting the quantile of the samples against the quantile of the disease sets, in the 25th - 75th percentile range. Function-constrained samples are shown in red, unconstrained samples in blue. Note that the samples are compared to the disease sets to make the Q-Q plots. The x-coordinate of a point in the Q-Q plot is the value of the given topological property in the disease set at a given percentile, whereas its y-coordinate is the average value of the topological property in the samples at the same percentile. Difference plots show the difference between the topological property of a disease set at a given quantile and the topological property of the samples at the same quantile. 1000 function-constrained and 1000 unconstrained samples have been generated for each disease set. The shaded areas encompass the interval between the bottom 5% and the top 95% of the values at a given quantile. See Additional file 1 Table S3 for empirical *p*-values assessing differences between topological features in disease and sample sets at the 25th, 50th and 75th percentiles.

Having determined that the functional composition of the disease sets can potentially play a role in the differences that set them apart from non-disease genes, we next test whether this is indeed the case by comparing disease sets with function-constrained samples generated as described above. We also consider functionally unconstrained samples as a baseline. The distributions of degree, clustering coefficient and betweenness centrality in the samples are compared against those of the disease sets in the 25th - 75th percentile range, using Q-Q plots (Figure 4); that is, for this range, we plot the quantile of the disease set against the quantile of the samples. The comparison reveals the noticeable effect of functionally constraining the samples, as for most disease set and topological feature pairings, the function-constrained samples are more similar to the disease sets than the unconstrained samples are. This is visually evident in Figure 4 with the Q-Q plots for the function-constrained samples closer to the diagonal (left columns), and their average difference from the disease sets closer to zero (right column). To quantify this, for each topological property and disease set, we measure the “area under the difference curve” as the sum across the interquartile range of the absolute value of the difference between the average value of the topological property in the samples and that in the disease set (Additional file 1 Table S2). We find that in 12 of the 15 cases, this area is smaller for function-constrained samples than for unconstrained samples. That is, with the exception of betweenness centrality for the OMIM and monogenic disease sets and degree for the OMIM disease set, all the other cases show smaller differences between samples and disease sets when function is taken into account.

The effect of comparing disease sets to functionally similar control sets can be striking. A particularly interesting example is provided by the clustering coefficient of Mendelian disorders genes. The distribution of clustering coefficient for the “monogenic” and “full OMIM” datasets would appear to be substantially lower than expected when looking at the unconstrained samples (all *p*-values < 0*.*05, see Additional file 1 Table S3), as observed by Cai et al. [22]. In contrast, by accounting for functional composition, the clustering coefficient does not significantly deviate from the values observed in the function-constrained samples at the 75th percentile (empirically determined *p*-values of 0.357 and 0.333 for the monogenic and OMIM disease sets, respectively) or has a median value larger than expected (*p* < 0*.*05 for the monogenic set and *p*<0*.*001for the OMIM set). This suggests that a large part of the differences for clustering coefficient in these two disease sets derive from their functional make-up.

In several cases, differences observed between disease sets and unconstrained samples are maintained once function is also considered. For example, a significant difference in degree exists between cancer over-expressed and under-expressed genes, even when accounting for their functional composition. Cancer over-expressed genes have larger than expected values at the 50th and 75th percentiles (*p*-values <0*.*01), whereas cancer under-expressed genes have lower than expected values (*p*-values <0*.*05). A similar trend was observed earlier, with the smaller set of genes differentially expressed in lung cancer [29].

In the case of betweenness centrality, Mendelian disorders genes, cancer mutated and cancer over-expressed genes show higher values than expected (even when accounting for their functional composition) across the whole interquartile range (all *p*-values <0*.*05), with cancer mutated genes exhibiting substantially larger values (*p*<0*.*001). In contrast, consistent with the results for degree, cancer under-expressed genes appear to be less central at the global level as well (*p*<0*.*05), although the differences are smaller when functional composition is taken into account.

We note that the results are robust with respect to the set of terms chosen, as sampling with a much larger set of 169 terms does not greatly affect the results (Additional file 1 Figure S3). We also repeat our analysis on three other networks, including a high-throughput network, derived from two sources [30,31]. Though there are some differences, the results obtained with these three networks are largely consistent with the results we report above (Additional file 1 Figures S4, S5 and S6 and Additional file 1 Tables S4-S14).

## Discussion

The function of a protein is to a large extent embedded in its interactions with other biomolecules. This suggests that the topological properties of a protein and its functional roles may be deeply intertwined. Our analysis of the human interactome shows that proteins belonging to different functional classes do indeed occupy topologically distinct regions of the network. This observation, which directly connects function to topology, has important repercussions for analyzing the topological properties of disease sets. In particular, disease genes are not an unbiased sample of the interactome, but are characterized by specific functional signatures that recapitulate the biology of the disorders.

We develop and apply a function-constrained sampling approach in order to compare disease sets with sample sets with a similar functional composition. A previous study of genes mutated in cancer [20] showed that they have a higher number of interactions, independent of their molecular functions; in that case, each gene was mapped to a single curated function and stratified sampling was applied to generate samples with the same functional properties. In contrast, our approach is fully automated and handles multiple functional annotations per gene. Further, an added benefit of our approach is that it automatically corrects for multifunctionality, an important confounding factor in network analysis [28].

By applying our function-constrained sampling approach, we find that the functional composition of the disease sets significantly affects the observed differences in the topological features of disease genes. The majority of cases show a smaller difference between disease and function-constrained samples, compared to unconstrained samples. In a few cases (e.g., the clustering coefficient for Mendelian disorders genes), the differences even lose statistical significance. At least two factors can be invoked to explain the observed effect of function constraining on the topological properties of disease genes. First, disease gene sets may be enriched or under-enriched in functional modules that have been studied more extensively than others, and therefore tend to have a larger number of known interactions. Controlling for function can therefore mitigate the effect of this particular type of study bias. A second factor directly involves the biology of protein interactions. For example, interactions tend to be more dense and clustered in protein complexes and more sparse in areas of the network implicated in primary metabolism. As observed by Goh et al. [1], Mendelian genes are under-enriched in essential genes, which tend to be clustered in protein complexes [17]. This could explain why Mendelian disorders genes do not appear less clustered than expected when factoring in their functional composition. Obviously, a combination of both factors can be at play. On the other hand, some disease sets still show differences that remain even when accounting for function. The high betweenness centrality in the case of cancer mutated genes, across the entire interquartile range, provides a particularly striking example (Figure 4c and Additional file 1 Figures S3-S6).

A caveat of most network analyses is that a larger fraction of interactions involving well-studied proteins and biological processes are likely to be known. Using only high-throughput interactions is one possible way to address the issue of study bias; however, it introduces other problems, such as a potentially higher amount of spurious interactions and an under-enrichment of interactions involving membrane proteins [32]. While obtaining complete and unbiased interactomes may very well lie beyond what can be achieved by purely computational means, our sampling strategy effectively deals with some of these bias-related problems at the level of functional modules.

The fact that certain topological properties are still distinct when factoring out function highlights the added value of topological information, especially in the potential context of identifying or prioritizing disease gene candidates. While approaches for identifying disease genes based on topological properties have been shown to be effective [21,33,34], incorporating the effects of function may lead to further performance improvements, as this better highlights the purely topological features that make disease genes distinct from other genes. Further, this type of combined functional-topological information may be especially useful in the context of integrative approaches for uncovering disease genes (e.g., [35,36]).

## Conclusions

As the number and types of disease gene sets continue to grow, network-based analysis of disease genes will continue to play a prominent role in attempts to characterize the complex interplay between network structure and pathological processes. In this paper, we study one of the most basic types of network analyses of disease genes—that of characterizing their topological properties. We show that in order to determine whether a set of disease genes is topologically distinct from other genes, it is necessary to explicitly consider biological function. We develop a computational framework for analyzing disease genes based upon a sampling strategy to generate control sets of genes functionally similar to a reference set. We apply this approach to study five disease gene sets, and demonstrate that in many cases topological features are distinct, in a disease set dependent manner, even when functional composition is considered.

## Methods

### Protein interaction data

For our primary analysis, we use a network derived from BioGrid v3.1.90 [26]. We extract all reported physical interactions, and remove all self-interactions along with all proteins with degree >500. The resulting BioGRID physical interaction network comprises nearly half of the human proteome, with 10042 proteins and 51756 interactions. To assess robustness, we also repeat the analysis on networks derived from two other sources and report the results in the Additional file 1 In particular, we also use the *Bossi* network [30], a comprehensive protein-protein interaction network compiled from several protein interaction databases (e.g., BIND [37], BioGRID [26] and HPRD [38]). Further, we use a high-throughput (*HT*) network derived from the HitPredict database [31]. In this database, interactions are called high-throughput if they are found in experiments determining more than 100 interactions. For the *HT* network, we only utilize high-throughput interactions that are also labelled as high confidence. We also consider a version of the HitPredict network that consists of all high-confidence interactions, whether they are high-throughput or not. As with the BioGrid network, we also remove self-edges and proteins with degree >500from the Bossi and HitPredict networks (See Additional file 1 Table S1 for the sizes of all networks used in our analysis).

### Disease genes

We consider five disease sets in three broad categories of genes involved in human disorders. Unless otherwise specified, our analysis is restricted to annotated genes, as we are considering the effects of functional composition. 

**(1) Genes with inherited mutations**. We extract the genes reported in the “morbid map” of the Online Mendelian Inheritance in Man (OMIM) catalogue [23], and map them onto the human protein interaction network. On the BioGRID network, this results in 1813 disease genes, 1580 of which have a functional annotation (“full OMIM” dataset). We also consider a subset of genes involved in monogenic disorders, obtained from Cai et al. [22]; this consists of 694 disease genes, 641 of which have a functional annotation (“monogenic disorders” dataset).

**(2) Genes with somatically acquired mutations in cancer**. We use the IntOGen repository (release 02) [24], which contains data extracted from the Catalogue of Somatic Mutations in Cancer (COSMIC) [39]. The COSMIC catalogue contains both data curated from the literature and the output from large-scale resequencing projects such as the Cancer Genome Project and The Cancer Genome Atlas project [40]. A total of 812 genes with at least one reported mutation in more than one cancer type can be mapped to the human protein interaction network, and 647 of these have a functional annotation (“cancer mutated” dataset).

**(3) Genes with significantly altered expression in cancer**. We extract the genes reported as significantly (*p*<0*.*001) over- or under-expressed in the IntOGen repository and map them to the human protein interaction network, obtaining a total of 6147 genes. Since 2389 genes are found to be both over- and under-expressed in different cancer types, we remove from the set of over-expressed genes those that are also found to be under-expressed in some cancer types and correspondingly for the under-expressed set, obtaining 1989 exclusively over-expressed genes (1359 with a functional annotation, “cancer over-expressed” set) and 1769 exclusively under-expressed genes (1169 with a functional annotation, “cancer under-expressed” set).

### Topological properties

We consider three widely studied topological properties of networks: degree, clustering coefficient, and betweenness centrality. The degree of a node in a network is simply defined as the number of edges incident upon that node. The clustering coefficient of a node is defined as the ratio of the number of triangles containing that node to the number of triples centered on it [41]; i.e., for a protein, this measures the number of interactions among its interactors, normalized by the maximum number of possible interactions. Betweenness centrality is defined as the sum over all other pairs of nodes *u* and *v* of the fraction of the shortest paths between *u* and *v* that pass through the node. These three topological properties are computed with the R package igraph[42].

### Functional annotation

Gene Ontology (GO) terms [43] in the “biological process” (BP) namespace are used throughout this work. We exclude electronic annotations and annotations inferred from physical interactions (evidence codes IEA and IPI, respectively). We use two sets of GO functional terms in our analysis. First, we consider the set of all GO BP terms that annotate more than 50 genes in human (169 terms). Next, we consider another set consisting of broader functional classes corresponding to “Informative Terms,” which are computed according to the method presented in Huang et al. [25]. The algorithm to extract informative terms selects terms that annotate ≥*n* of genes, and retains only those terms whose children annotate <*n*genes. A reasonably large *n* is chosen to select sufficiently broad terms, while terms that annotate many genes simply because of their children terms are automatically discarded. With *n*=500, we obtain a set of 46 informative terms.

For the primary results reported in the paper, we use the set of 46 informative functional terms, with analysis performed on the more comprehensive sets of terms reported in the Additional file 1 The 46 informative terms are used for visualization in Figure 2, and for generating samples (see Section “Function-constrained sampling algorithm”). GO enrichment analysis on sets of genes is performed using the hypergeometric test, with multiple hypothesis testing corrections via the Bonferroni correction. Enrichment analysis is always performed on the *full set* of GO terms annotating the genes and all of their ancestors in the GO graph (i.e., not just informative terms).

### Function-constrained sampling algorithm

We develop and apply an iterative sampling scheme to generate non-disease samples with a functional composition similar to that of the disease sets. More precisely, we aim to minimize the distance between the per-term frequency distribution in a disease set and the distribution in a random sample of non-disease genes. We note that a simple approach that selects (for each informative term) a number of genes equal to the term frequency in the disease set is not effective in minimizing functional biases, because genes are often annotated with multiple terms. That is, such a stratified sampling approach is not applicable and would result in a set of genes with a different functional composition than the original disease set. In contrast, our iterative sampling approach takes this fact into account. The algorithm directly compares the term frequency in the disease set against the term frequency in the samples, and attempts to minimize the difference.

Let *T*={*t*_1_,*t*_2_,…,*t*_*N*_} be the set of GO terms we are considering. The per-term frequency distribution *F* in a set *G* of genes is defined as:

(1) $$F^{G}=\langle f_{1}^{G},f_{2}^{G},\ldots ,f_{N}^{G}\rangle$$

where *f*_*i*_ is the number of genes in *G* that have annotation *t*_*i*_. For two sets of genes *X* and *Y *, we define the “functional distance” between the per-term frequency distributions as:

(2) $$\mathrm{dist}(F^{X},F^{Y})=\overset{N}{\underset{i=1}{\sum }}{\left(f_{i}^{X}-f_{i}^{Y}\right)}^{2}$$

Then, letting *D* denote the reference set of disease genes, *B* the background (or control) set of non-disease genes, and *S* the sample to extract from *B*, our function-constrained sampling scheme can be summarized by the following steps: 

1. Initialize the sample set *S* by randomly drawing from the non-disease set *B* a number of genes equal to that of the disease set *D*

2. **Repeat**

a) randomly pick a gene *g*_*i*_from *S* and a gene *g*_*j*_from *B*−*S*(i.e., from the pool of non-disease genes *not* in the current sample)

b) *S*^*new*^←*S*−{*g*_*i*_} + {*g*_*j*_}

c) **if***dist*(*F*^*D*^,*F*^*S*^_*new*_)<*dist*(*F*^*D*^,*F*^*S*^)**then***S*←*S*^*new*^

**until***dist*(*F*^*D*^,*F*^*S*^)=0or a chosen maximum number of steps has been reached.

Our implementation of the sampling algorithm (see http://compbio.cs.princeton.edu/fcsampling for source code available under a GNU public license) also includes a variant that uses a Metropolis criterion to accept or reject moves. Instead of rejecting a move that increases the distance from the target distribution, the algorithm accepts it with an exponentially decaying probability *p*:

$$p=\min(\exp(-c[\mathrm{dist}(F^{D},F^{S_{\mathrm{new}}})-\mathrm{dist}(F^{D},F^{S})]),1)$$

As *c*>0 is increased, the probability of accepting a “bad” move becomes smaller. We tested the Metropolis variant for increasing values of *c*, and compared it against the initial greedy algorithm by measuring the variability between samples, the value of *dist*(*F*^*D*^,*F*^*S*^), and convergence times. In preliminary testing, we found no advantage in using the Metropolis criterion (Additional file 1 Figure S2), and thus the reported results use the initially described greedy approach.

### Generating non-disease sets that are functionally similar to the disease sets

We use the sampling scheme outlined above, with a maximum of 10^5^ steps. We note that this threshold is robust, as in practice the procedure converges within a much smaller number of steps (∼10^4^ steps). We generate 1000 sets of non-disease genes functionally similar to the corresponding five disease sets. The pool of non-disease genes for the “monogenic disorders”, “full OMIM” and “cancer mutated” sets is obtained by subtracting from the annotated genes in the human network all genes with a reported mutation in any of the three disease sets. For the “cancer over-expressed” and “cancer under-expressed” genes, we use the annotated genes not reported as significantly (*p*<0*.*001) over-expressed and under-expressed by IntOGen, respectively.

### Comparing distributions

To compare and visualize the distribution of the topological properties of the samples against those of the disease sets, we use Q-Q plots. Q-Q plots are obtained by plotting the quantile of one distribution against the same quantile of the other, for a given range of quantiles. We consider the interquartile range—i.e., the interval encompassing the 25th percentile and the 75th percentile of each property in the gene sets. If the distribution of values in the samples is similar to that in the disease sets, the points will lie along the diagonal. In contrast, if the disease sets have higher (respectively, lower) values compared to the samples, the points will lie below (respectively, above) the diagonal. Since our analysis comprises 1000 constrained and 1000 unconstrained samples, we compute average values at each quantile and show confidence intervals as shaded areas around the average. The boundaries of the shaded areas are given by the 5th and 95th percentiles of the values in the samples. Further, to facilitate the comparison, we plot the difference between the values in the disease sets and the values in the samples over the interquartile range. We empirically estimate *p*-values at the 25th, 50th and 75th percentiles by counting the fraction of samples with a more extreme value than that observed in the disease set at the same percentile.

## Competing interests

The authors declare that they have no competing interests.

## Authors’ contributions

DG and MS conceived the study, designed the computational methods, analyzed the data, and wrote the manuscript. DG developed and tested the algorithms, and performed all the computational experiments. All authors have read and approved the final manuscript.

## Funding

American-Italian Cancer Foundation Postdoctoral Fellowship to D.G.; National Science Foundation (ABI-0850063 to M.S., in part); National Institutes of Health (GM076275 to M.S., in part); National Institute of Health Center of Excellence (grant P50 GM071508 to David Botstein, in part).

## Supplementary Material

Additional file 1 **Supplementary Figures and Tables. **Additional figures and tables containing additional information on the method and results obtained with a larger set of informative terms and on other networks. Click here for file
