# Supplementary material for: Disentangling function from topology to infer the network properties of disease genes
Source: BMC Syst Biol. 2013 Jan 16;7:5. doi: 10.1186/1752-0509-7-5 (PMC3614482; doi:10.1186/1752-0509-7-5)
Supplement: Additional file 1 — Supplementary Figures and Tables. Additional figures and tables containing additional information on the method and results obtained with a larger set of informative terms and on other networks. [file 1752-0509-7-5-S1.pdf]

	number of proteins	number of interactions
BioGRID	10042	51756
Bossi	10229	80651
HitPredict (HT/HC)	7767	35209
HitPredict (Full/HC)	8302	42015

Table 1. Network statistics. For each of the four networks used in this study, we give the number of proteins and the number of interactions amongst them.

		**Degree**	**Clustering**	**Betweenness**
monogenic	constr	**31.881** (1.5e-24)	**0.265** (0.0e+00)	80943.442 (3.8e-200)
	unconstr.	39.939	1.615	53750.948
OMIM	constr	48.445 (0.0e+00)	**0.244** (0.0e+00)	103209.311 (4.7e-163)
	unconstr.	11.474	0.972	90710.830
cancerMut	constr	**254.584** (0.0e+00)	**0.806** (6.5e-62)	**324639.680** (0.0e+00)
	unconstr.	314.122	0.923	390282.497
cancerOverExpr	constr	**63.300** (0.0e+00)	**0.294** (1.6e-271)	**48880.825** (3.6e-280)
	unconstr.	124.901	0.635	80852.172
cancerUnderExpr	constr	**52.049** (0.0e+00)	**0.121** (5.8e-303)	**43267.235** (9.8e-287)
	unconstr.	112.447	0.705	78833.287

Table 2. Average area under the difference plots (see Figure 4 in main paper). For each percentile in the interquartile range, we compute the absolute value of the difference in the value of the topological feature between the disease set and the average in the sample sets, and then sum across the interquartile range. In 12 out of 15 cases, these values are smaller for constrained samples than they are for unconstrained samples, indicating that in these cases the the topological features of the constrained samples are closer to the disease sets than those of the unconstrained samples. We compare the distributions of area under the difference plots for the function-constrained and unconstrained samples using the Wilcoxon's rank sum test and report the p-values in parenthesis next to the value for constrained samples. The values for the constrained samples are bolded whenever found significantly smaller ($p < 0.05$) than the values for the unconstrained samples.

		**Degree**			**Clustering**			**Betweenness**		
		25%	50%	75%	25%	50%	75%	25%	50%	75%
monogenic	constr.	0.982	0.224	0.304	—	0.038	0.357	0.000	0.000	0.000
	unconstr.	0.970	0.405	0.041	—	0.001	0.000	0.109	0.006	0.008
OMIM	constr.	1.000	0.000	0.000	—	0.000	0.333	0.000	0.000	0.000
	unconstr.	1.000	0.656	0.043	—	0.028	0.000	0.000	0.000	0.000
cancerMut	constr.	0.000	0.000	0.000	0.000	0.000	0.401	0.000	0.000	0.000
	unconstr.	0.000	0.000	0.000	0.000	0.000	0.227	0.000	0.000	0.000
cancerOverExpr	constr.	0.351	0.000	0.004	—	0.004	0.469	0.000	0.000	0.021
	unconstr.	0.000	0.000	0.000	—	0.000	0.230	0.000	0.000	0.000
cancerUnderExpr	constr.	1.000	0.005	0.020	—	0.175	0.440	0.000	0.000	0.016
	unconstr.	0.707	0.000	0.000	—	0.000	0.002	0.000	0.000	0.000

Table 3. Uncorrected empirical p-values, showing the significance of the difference between disease sets and samples (full BioGRID network, Informative terms). We use 1000 function-constrained and unconstrained samples and estimate the fraction of the 1000 that have more extreme values than the disease set at the 25th, 50th and 75th percentiles. Dashes indicate cases where the values for the disease set and all the samples are 0.

		**Degree**	**Clustering**	**Betweenness**
monogenic	constr	35.752 (2.6e-76)	**0.272** (0.0e+00)	69049.337 (5.3e-164)
	unconstr.	25.322	1.401	48685.396
OMIM	constr	56.622 (0.0e+00)	**0.326** (0.0e+00)	100207.350 (5.2e-110)
	unconstr.	20.108	0.844	91665.699
cancerMut	constr	**219.299** (0.0e+00)	**0.714** (1.5e-242)	**298085.415** (0.0e+00)
	unconstr.	310.151	0.969	373928.391
cancerOverExpr	constr	**43.201** (0.0e+00)	**0.279** (9.7e-245)	**30911.929** (1.7e-303)
	unconstr.	102.016	0.548	64035.740
cancerUnderExpr	constr	**39.741** (0.0e+00)	**0.167** (9.5e-302)	**32561.069** (3.6e-303)
	unconstr.	94.616	0.724	67643.124

Table 4. **Area under the difference plots (BioGRID network, 25th - 75th percentile, all terms).**

		**Degree**	**Clustering**	**Betweenness**
monogenic	constr	**269.420** (1.7e-61)	**1.871** (8.5e-305)	**1576716.468** (1.1e-76)
	unconstr.	334.266	4.506	1886125.121
OMIM	constr	**270.317** (2.1e-23)	**0.813** (0.0e+00)	**1448335.491** (6.0e-301)
	unconstr.	303.395	2.936	1800387.363
cancerMut	constr	**849.024** (0.0e+00)	**2.702** (6.5e-165)	**2539088.477** (1.1e-307)
	unconstr.	1249.390	3.734	3572482.428
cancerOverExpr	constr	**269.325** (5.7e-283)	2.142 (9.9e-06)	714510.996 (3.5e-05)
	unconstr.	409.109	2.047	578670.292
cancerUnderExpr	constr	**185.315** (5.9e-306)	**0.989** (2.9e-63)	**202111.157** (1.4e-135)
	unconstr.	355.150	1.311	623950.033

Table 5. **Area under the difference plots (BioGRID Full network, whole distribution, informative terms)**. The whole distribution is considered, instead of the 25th-75th percentile range as in Table S2.

		**Degree**	**Clustering**	**Betweenness**
monogenic	constr	**45.184** (9.4e-119)	**0.857** (0.0e+00)	126300.285 (2.2e-166)
	unconstr.	64.660	3.128	99172.000
OMIM	constr	48.626 (2.2e-193)	**1.144** (0.0e+00)	120300.116 (4.1e-45)
	unconstr.	35.267	2.939	113664.774
cancerMut	constr	**194.382** (6.8e-225)	**1.238** (0.0e+00)	**275542.322** (3.9e-294)
	unconstr.	246.691	2.523	337117.670
cancerOverExpr	constr	**117.403** (0.0e+00)	**1.157** (0.0e+00)	**39602.370** (2.1e-282)
	unconstr.	207.455	2.476	79597.078
cancerUnderExpr	constr	**76.040** (0.0e+00)	**1.073** (0.0e+00)	**56693.246** (4.0e-298)
	unconstr.	171.433	2.765	102528.439

Table 6. **Area under the difference plots (Bossi network, 25th - 75th percentile, informative terms)**

		**Degree**	**Clustering**	**Betweenness**
monogenic	constr	56.142 (1.4e-27)	**0.737** (0.0e+00)	134562.445 (5.7e-304)
	unconstr.	49.355	2.757	90571.337
OMIM	constr	66.754 (0.0e+00)	**0.854** (0.0e+00)	138222.652 (1.9e-293)
	unconstr.	27.999	2.708	116443.527
cancerMut	constr	**210.743** (4.1e-274)	**0.796** (0.0e+00)	**273304.868** (0.0e+00)
	unconstr.	264.334	2.320	331739.155
cancerOverExpr	constr	**84.614** (0.0e+00)	**0.576** (0.0e+00)	**25683.587** (0.0e+00)
	unconstr.	181.792	2.189	69806.237
cancerUnderExpr	constr	**52.443** (0.0e+00)	**0.741** (0.0e+00)	**32133.205** (0.0e+00)
	unconstr.	154.195	2.705	86775.846

Table 7. **Area under the difference plots (Bossi network, 25th - 75th percentile, all terms)**

		Degree	Clustering	Betweenness
monogenic	constr	**393.612** (0.0e+00)	**3.889** (0.0e+00)	573727.029 (2.1e-01)
	unconstr.	777.877	9.341	603572.290
OMIM	constr	**420.469** (1.4e-190)	**4.156** (0.0e+00)	**680539.251** (1.9e-290)
	unconstr.	517.556	8.462	719215.473
cancerMut	constr	**449.293** (1.6e-18)	**6.478** (0.0e+00)	**1675486.852** (0.0e+00)
	unconstr.	450.797	9.545	2640773.202
cancerOverExpr	constr	**406.059** (0.0e+00)	**1.962** (0.0e+00)	**481943.463** (5.9e-61)
	unconstr.	834.754	4.373	696409.173
cancerUnderExpr	constr	**444.953** (0.0e+00)	**2.850** (0.0e+00)	**566590.538** (1.2e-133)
	unconstr.	987.238	6.044	949630.946

Table 8. **Area under the difference plots (Bossi network, whole distribution, informative terms)**

		Degree	Clustering	Betweenness
monogenic	constr	**10.492** (0.0e+00)	**0.359** (1.3e-211)	36515.100 (8.4e-104)
	unconstr.	55.774	0.771	21939.045
OMIM	constr	**15.205** (6.5e-03)	**0.311** (1.5e-229)	**47668.962** (1.2e-11)
	unconstr.	15.664	0.542	49635.937
cancerMut	constr	**174.732** (2.1e-237)	0.528 (1.6e-04)	**171190.850** (1.3e-274)
	unconstr.	206.454	0.466	204305.710
cancerOverExpr	constr	**66.655** (0.0e+00)	**0.429** (2.5e-303)	**32301.476** (7.9e-229)
	unconstr.	102.407	0.730	51398.491
cancerUnderExpr	constr	**51.658** (0.0e+00)	**0.420** (6.1e-297)	**27547.950** (6.6e-244)
	unconstr.	100.376	0.940	51960.576

Table 9. **Area under the difference plots (HitPredict HT/HC, 25th - 75th percentile, informative terms)**

		Degree	Clustering	Betweenness
monogenic	constr	**11.078** (2.2e-299)	**0.278** (1.7e-233)	35564.413 (4.6e-113)
	unconstr.	42.007	0.690	23065.503
OMIM	constr	33.837 (0.0e+00)	**0.318** (1.0e-99)	55470.005 (2.4e-01)
	unconstr.	10.947	0.427	54968.662
cancerMut	constr	**152.808** (0.0e+00)	**0.367** (1.4e-134)	**159653.579** (0.0e+00)
	unconstr.	209.638	0.457	198565.208
cancerOverExpr	constr	**51.047** (0.0e+00)	**0.357** (0.0e+00)	**20504.216** (1.3e-303)
	unconstr.	93.638	0.667	45519.636
cancerUnderExpr	constr	**37.432** (0.0e+00)	**0.255** (0.0e+00)	**19861.252** (3.3e-293)
	unconstr.	88.803	0.859	45745.235

Table 10. **Area under the difference plots (HitPredict HT/HC, 25th - 75th percentile, all terms)**

		Degree	Clustering	Betweenness
monogenic	constr	**127.432** (5.7e-181)	**0.972** (5.3e-283)	660506.131 (4.1e-01)
	unconstr.	219.654	2.763	645083.736
OMIM	constr	**89.578** (7.8e-290)	**0.659** (0.0e+00)	**329548.045** (0.0e+00)
	unconstr.	208.110	2.400	866396.372
cancerMut	constr	**512.178** (0.0e+00)	**2.038** (1.3e-204)	**970541.785** (1.2e-285)
	unconstr.	854.108	3.102	1904702.053
cancerOverExpr	constr	**262.498** (3.5e-135)	1.196 (7.0e-05)	684309.173 (2.5e-36)
	unconstr.	307.496	1.091	549592.607
cancerUnderExpr	constr	**157.672** (4.0e-273)	**1.092** (3.1e-146)	**402879.237** (7.4e-72)
	unconstr.	309.937	1.963	633603.131

Table 11. **Area under the difference plots (HitPredict HT/HC, whole distribution, informative terms)**

		Degree	Clustering	Betweenness
monogenic	constr	**20.585** (3.0e-186)	**0.417** (6.1e-180)	71139.409 (2.9e-183)
	unconstr.	46.039	0.875	49560.227
OMIM	constr	**18.355** (1.9e-43)	**0.260** (0.0e+00)	63621.741 (3.3e-02)
	unconstr.	21.049	0.709	62696.523
cancerMut	constr	**215.323** (1.7e-301)	**0.707** (3.0e-44)	**179646.929** (0.0e+00)
	unconstr.	268.268	0.775	226051.050
cancerOverExpr	constr	**74.521** (0.0e+00)	**0.481** (6.4e-306)	**34704.895** (1.4e-263)
	unconstr.	124.498	0.893	58235.376
cancerUnderExpr	constr	**62.488** (0.0e+00)	**0.577** (3.1e-294)	**38975.349** (6.7e-277)
	unconstr.	123.181	1.209	69523.613

Table 12. **Area under the difference plots (HitPredict Full/HC, 25th - 75th percentile, informative terms)**

		Degree	Clustering	Betweenness
monogenic	constr	**21.238** (1.5e-87)	**0.175** (4.7e-307)	61563.627 (5.4e-118)
	unconstr.	35.042	0.938	47896.571
OMIM	constr	40.259 (0.0e+00)	**0.325** (3.5e-301)	65207.651 (9.5e-01)
	unconstr.	13.240	0.675	65091.417
cancerMut	constr	**186.600** (0.0e+00)	**0.550** (7.5e-209)	**167295.110** (0.0e+00)
	unconstr.	270.628	0.745	221327.000
cancerOverExpr	constr	**58.528** (0.0e+00)	**0.402** (0.0e+00)	**24851.049** (0.0e+00)
	unconstr.	113.489	0.914	52108.662
cancerUnderExpr	constr	**49.653** (0.0e+00)	**0.584** (0.0e+00)	**29727.098** (2.0e-288)
	unconstr.	108.471	1.236	58590.922

Table 13. **Area under the difference plots (HitPredict Full/HC, 25th - 75th percentile, all terms)**

		Degree	Clustering	Betweenness
monogenic	constr	**127.432** (5.7e-181)	**0.972** (5.3e-283)	660506.131 (4.1e-01)
	unconstr.	219.654	2.763	645083.736
OMIM	constr	**89.578** (7.8e-290)	**0.659** (0.0e+00)	**329548.045** (0.0e+00)
	unconstr.	208.110	2.400	866396.372
cancerMut	constr	**512.178** (0.0e+00)	**2.038** (1.3e-204)	**970541.785** (1.2e-285)
	unconstr.	854.108	3.102	1904702.053
cancerOverExpr	constr	**262.498** (3.5e-135)	1.196 (7.0e-05)	684309.173 (2.5e-36)
	unconstr.	307.496	1.091	549592.607
cancerUnderExpr	constr	**157.672** (4.0e-273)	**1.092** (3.1e-146)	**402879.237** (7.4e-72)
	unconstr.	309.937	1.963	633603.131

Table 14. **Area under the difference plots (HitPredict Full/HC, whole distribution, informative terms)**

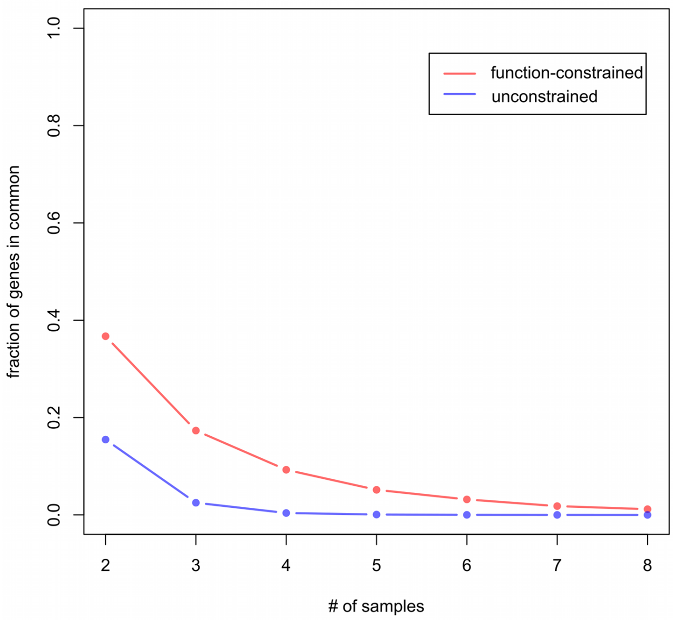

Figure 1. The function-constrained control samples are sufficiently diverse. Overlap amongst the samples as an increasing number of function-constrained (red, using Informative Terms) or unconstrained (blue) samples are generated. As a function of the number of function-constrained (respectively, unconstrained) samples, we plot the fraction of genes shared amongst all the samples. The results shown are the average over generating samples 100 times, using the monogenic disorders set as the reference set; error bars are plotted but are too small to be visible.

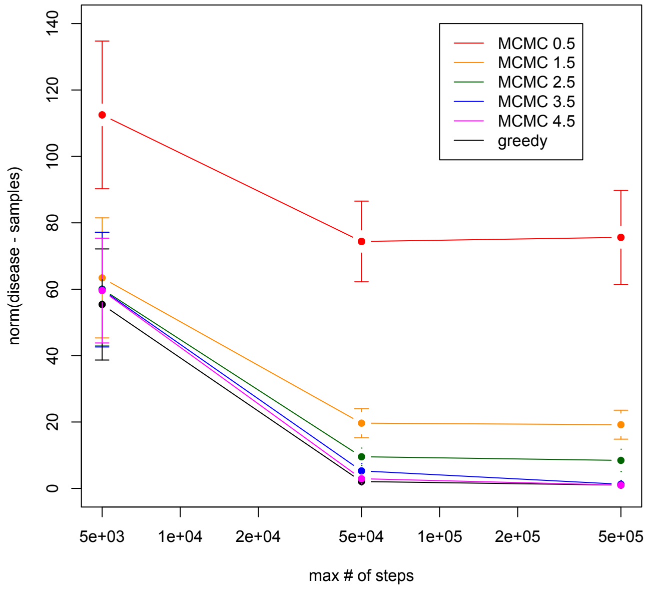

Figure 2. Comparison of the MCMC and greedy sampling approaches. To compare the MCMC against the greedy sampling strategy, we generated constrained samples with the MCMC algorithm, varying the parameter c from 0.5 to 4.5, in 1.0 increments and capping the maximum number of steps to 50^3, 50^4 and 50^5. We then compared the Euclidean norm between the distribution of informative term functional annotations in the disease set versus those found in the samples. The results indicate that for $c{\sim}4.5$ the MCMC reaches the same convergence times of the greedy algorithm.

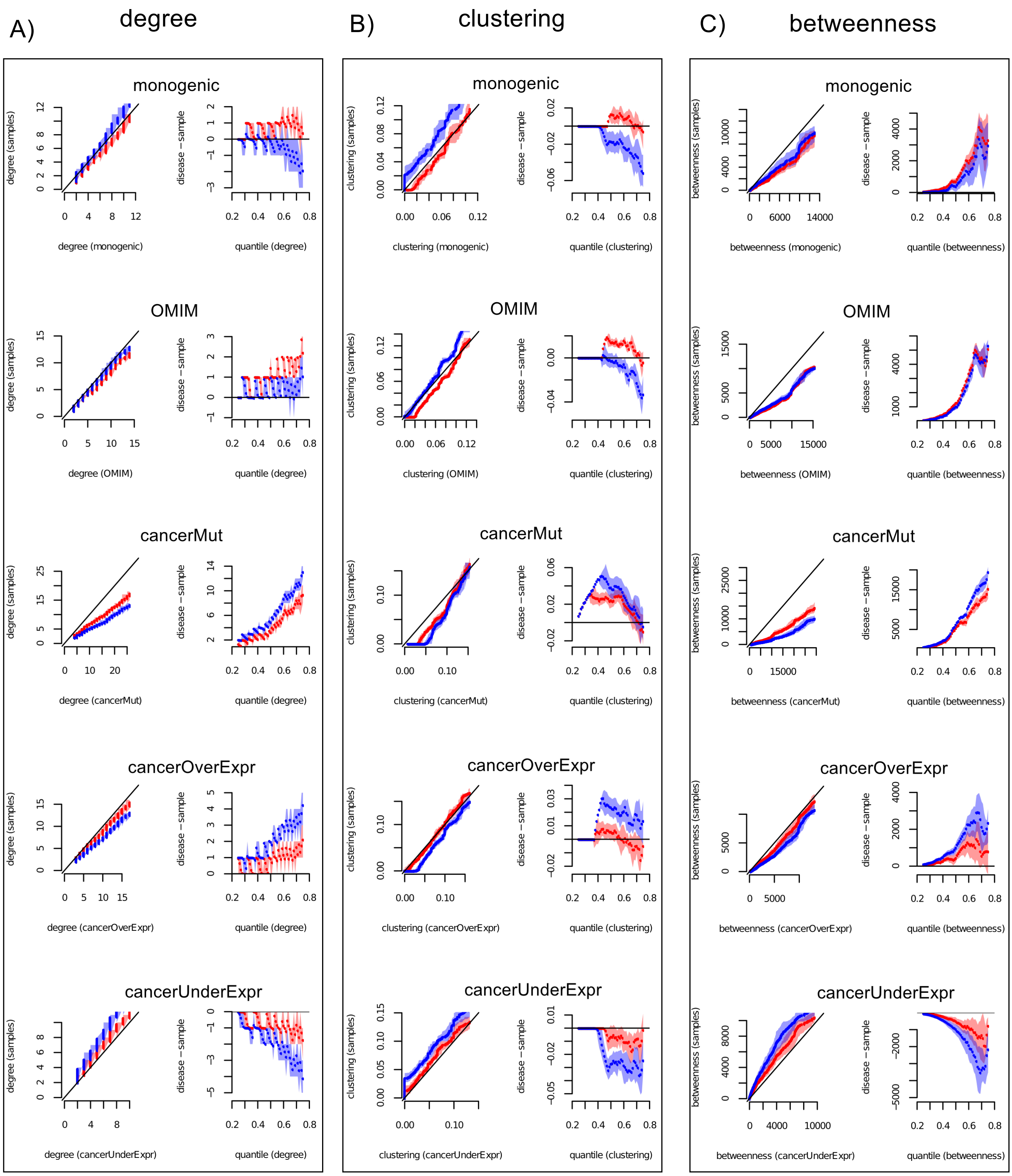

Figure 3. BioGRID Full, all terms. Q-Q plots (left-most columns) and difference plots (right-most columns) for degree (A), clustering coefficient (B) and betweenness centrality (C). Function-constrained samples are shown in red, unconstrained samples in blue. The Q-Q plots are obtained by plotting the quantile of the samples against the quantile of the disease sets, in the 25th - 75th percentile range. Difference plots show the difference between the topological property of a disease set at a given quantile and the topological property of the samples at the same quantile. 1000 function-constrained and 1000 unconstrained samples have been generated for each disease set. The shaded areas encompass the interval between the bottom 5% and the top 95% of the values at a given quantile.

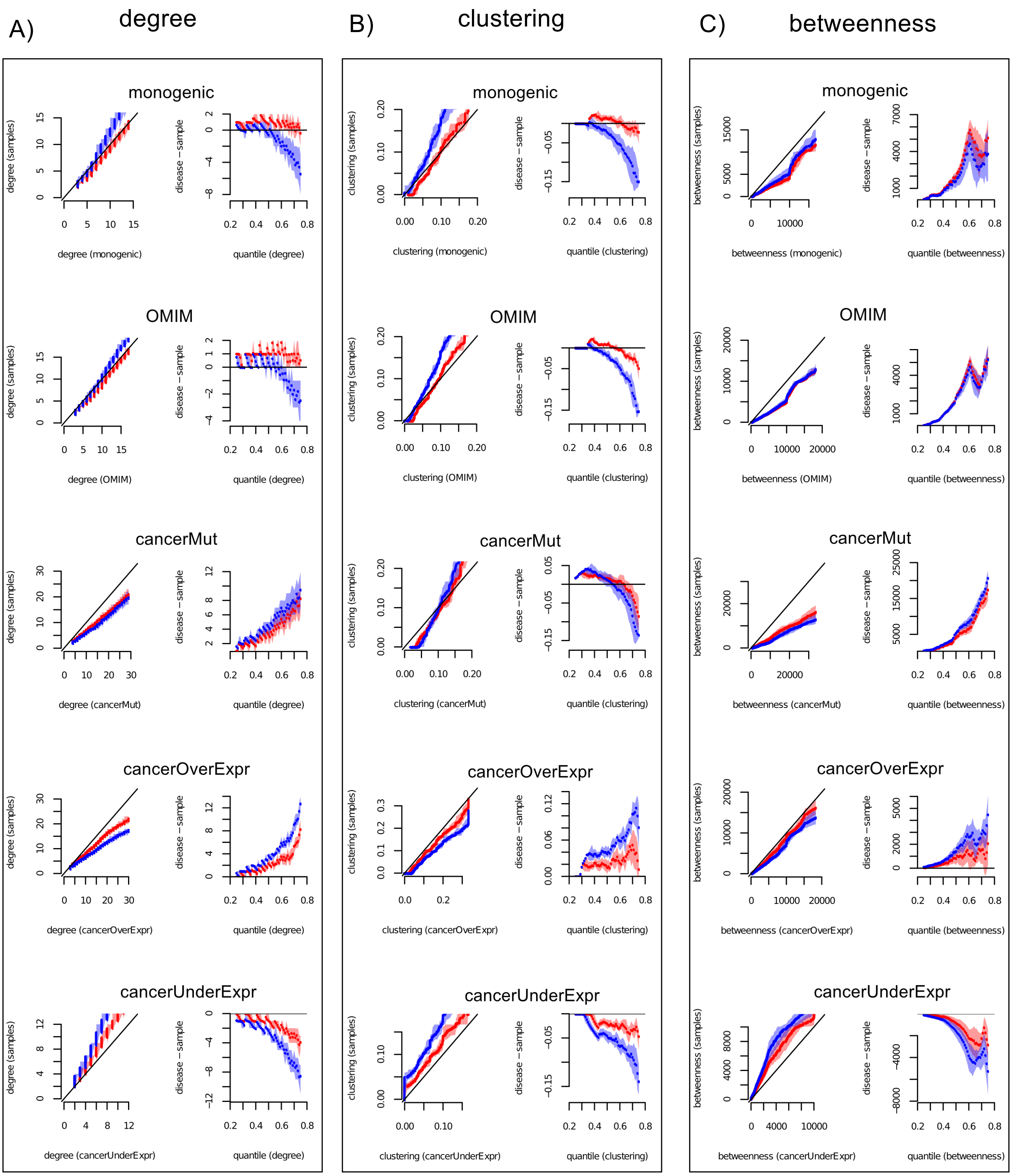

Figure 4. Bossi network, Informative Terms. See caption for Supplementary Figure 3.

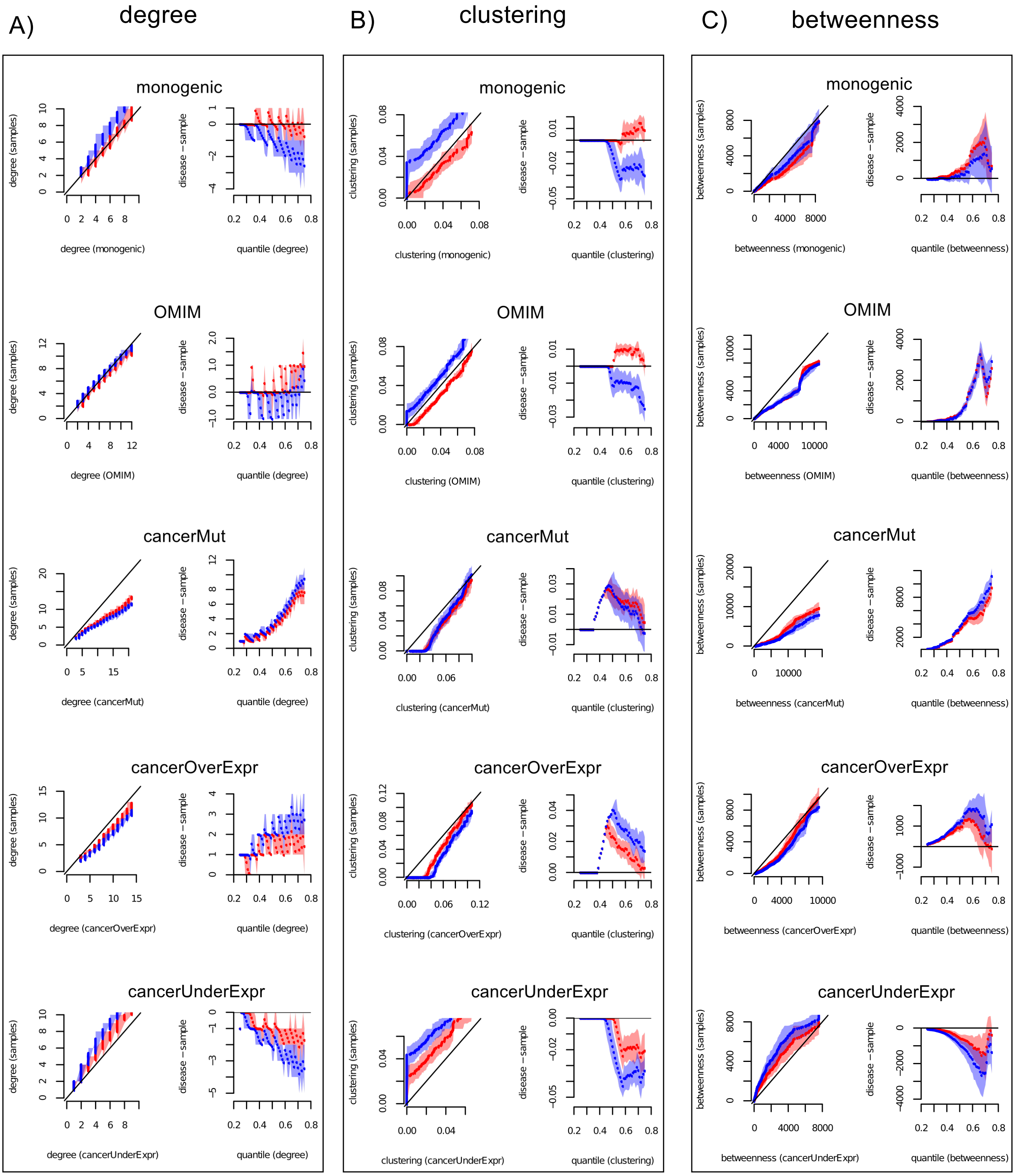

Figure 5. HitPredict network (High-throughput, High Confidence), Informative Terms. See caption for Supplementary Figure 3.

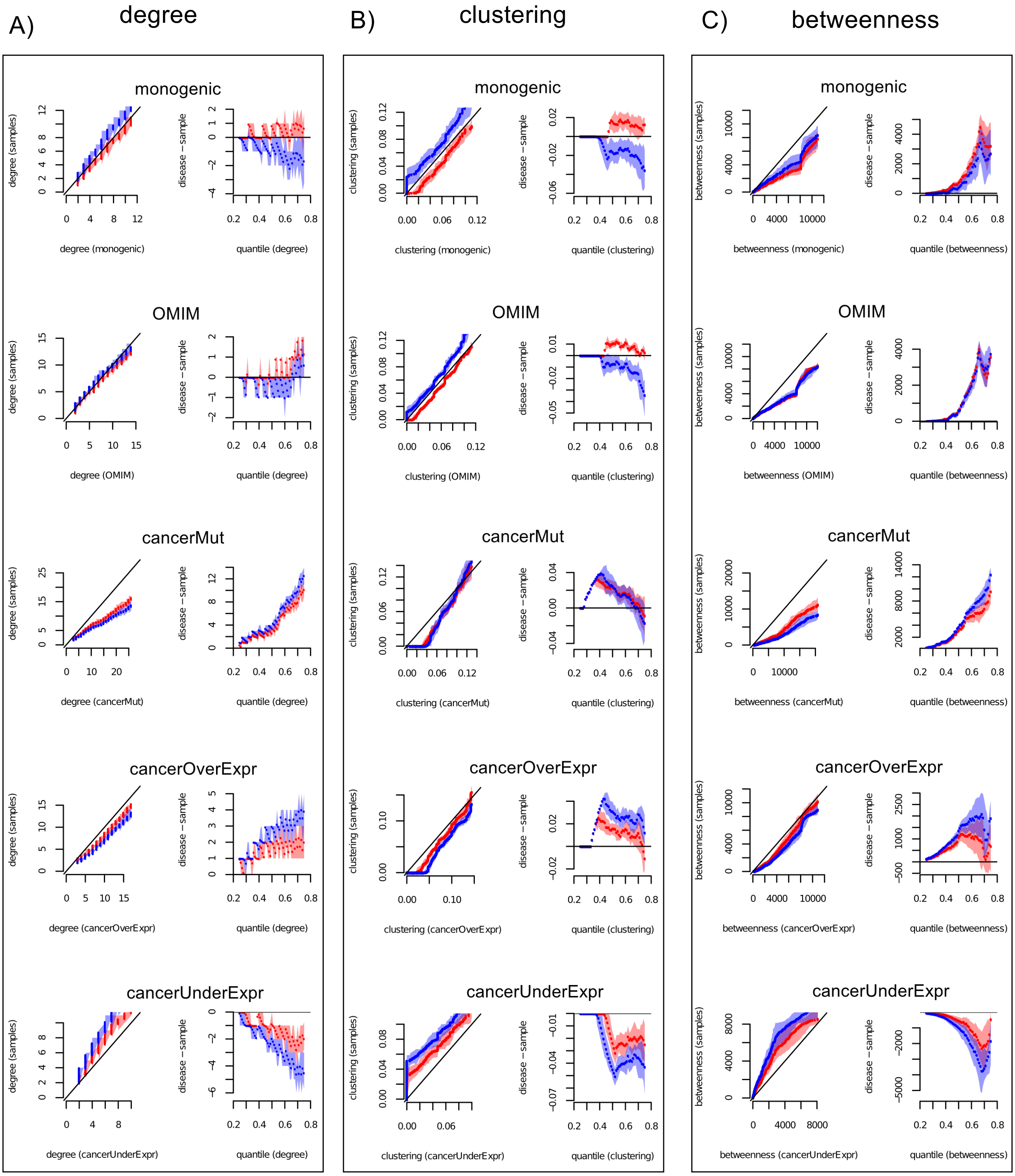

Figure 6. HitPredict network (Full, High Confidence), Informative Terms. See caption for Supplementary Figure 3.

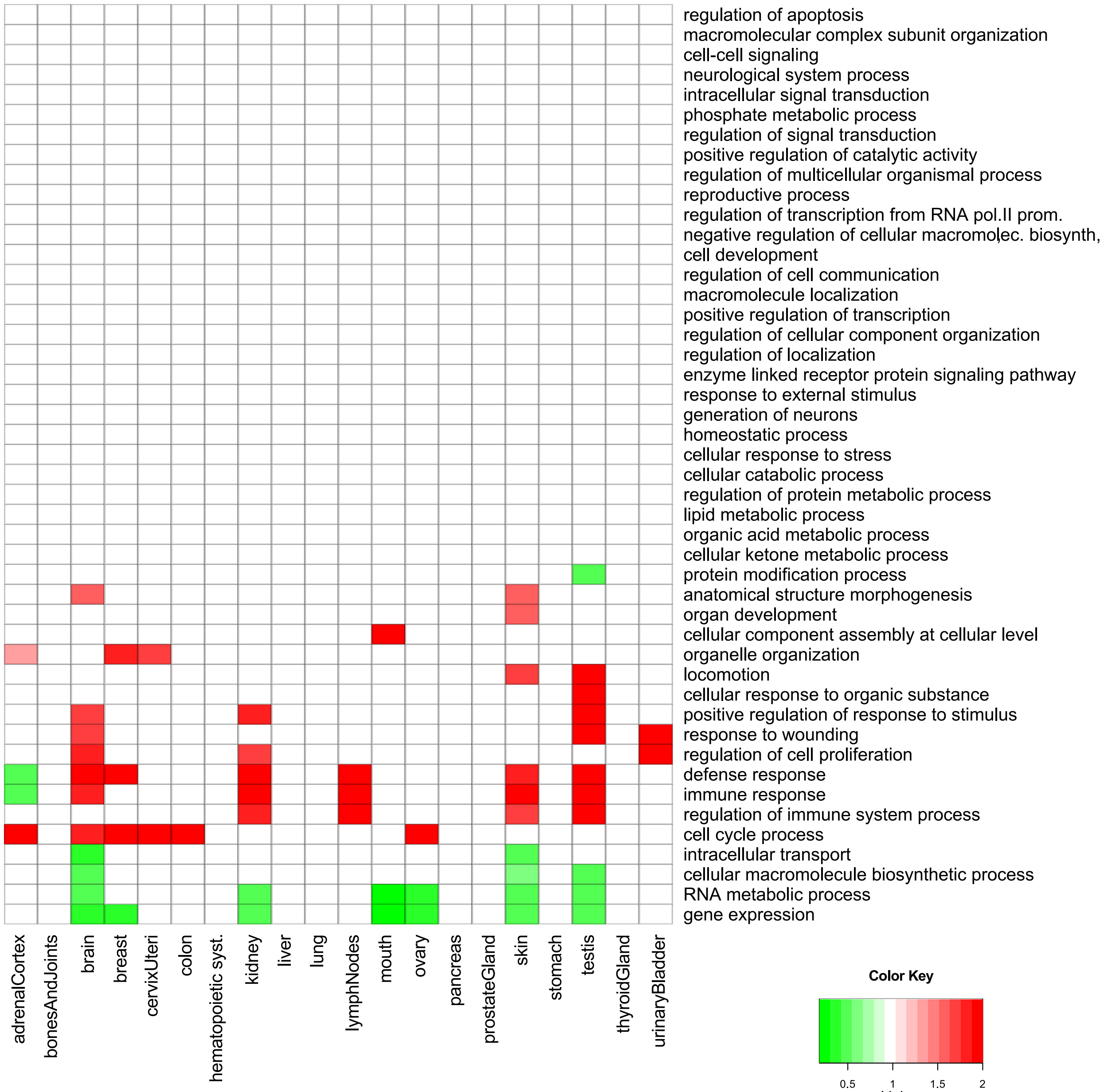

Figure 7. GO term enrichment and depletion in over-expressed genes, subdivided by anatomical region (IntOGen classification). For each anatomical region, significantly enriched and under-enriched functions are shown in red and green, respectively ($p < 0.05$, Bonferroni-corrected hypergeometric test). The fold enrichment or depletion for each disease set and term is plotted using a red-green gradient and calculated as the ratio of the fraction of genes in the disease set that are annotated by that term to the fraction of all annotated genes that include that term.

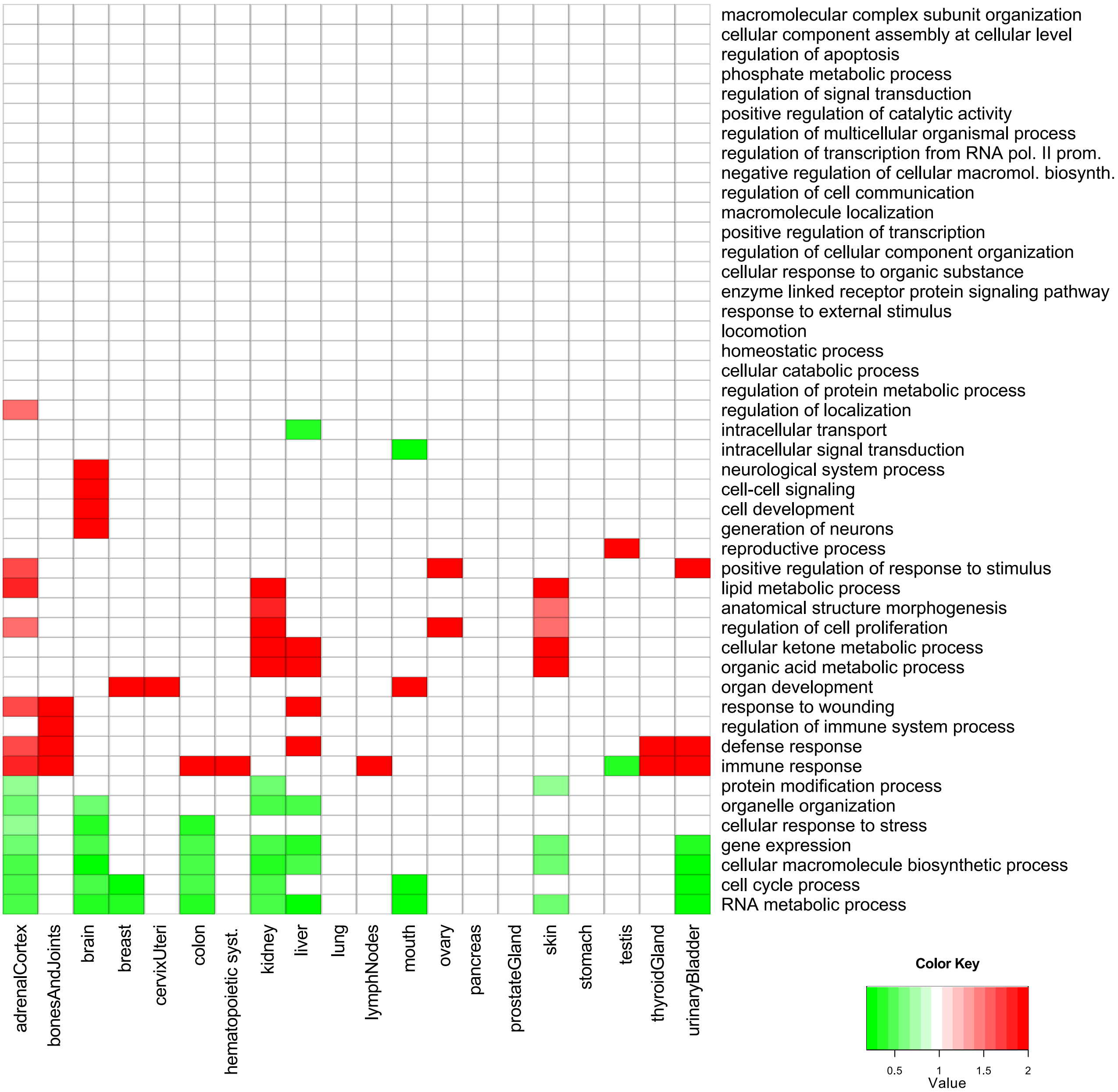

Figure 8. GO term enrichment and depletion in under-expressed genes, subdivided by anatomical region (IntOGen classification). For each anatomical location, significantly enriched and under-enriched functions are shown in red and green, respectively ($p < 0.05$, Bonferroni-corrected hypergeometric test). The fold enrichment or depletion for each disease set and term is plotted using a red-green gradient and calculated as the ratio of the fraction of genes in the disease set that are annotated by that term to the fraction of all annotated genes that include that term.
